# Supplementary material for: Complexome profiling of the Chlamydomonas psb28 mutant reveals TEF5 as an early PSII assembly factor
Source: Plant Cell. 2025 Mar 17;37(6):koaf055. doi: 10.1093/plcell/koaf055 (PMC12164590; doi:10.1093/plcell/koaf055)
Supplement: koaf055_Supplementary_Data [file koaf055_supplementary_data.zip › psb28_interactive.zip!/plots/intensity/qualityPlots/peptides/CGL74_Cre12.g554103.t1.1.html]

### Cre12.g554103.t1.1

Trivial name: CGL74  
SeqCoverage [median]: 18.70  
Mass: 60.77 kDa  
  
 

| Sequence | MissedCleavage | Mass [Da] | wtIntensity | muIntensity | Score |
| --- | --- | --- | --- | --- | --- |
| AAGGGAAASTISDPALR | 0 | 1484.76 | 3415.77 | 3283.04 | 152.47 |
| AIADATPTAPQLPDNN | 0 | 1607.78 | 1466.53 | 3974.24 | 153.34 |
| FYAPASK | 0 | 782.40 | 0.00 | 1344.39 | 69.43 |
| GHEQDALATYEELIK | 0 | 1715.84 | 622.35 | 3120.50 | 51.15 |
| LLGESSLLSQQSR | 0 | 1416.76 | 4935.79 | 11595.14 | 178.67 |
| LYPGAMGLIPGQAQQQR | 0 | 1826.95 | 0.00 | 504.91 | 48.44 |
| VALYEDQIK | 0 | 1077.57 | 0.00 | 950.20 | 62.00 |
| YAGVRPAQSPPDAIEAK | 1 | 1768.91 | 8433.30 | 13251.88 | 104.44 |
|  |  |  | 18873.7 | 38024.3 |  |
